# Exposure to perfluorooctanoic acid (PFOA) decreases neutrophil migration response to injury in zebrafish embryos

**DOI:** 10.1186/s13104-020-05255-3

**Published:** 2020-08-31

**Authors:** Alison M. Pecquet, Andrew Maier, Susan Kasper, Saulius Sumanas, Jagjit Yadav

**Affiliations:** 1 Department of Environmental and Public Health Sciences, University of Cincinnati College of Medicine, 160 Panzeca Way, Cincinnati, OH 45267 USA; 2 Cardno ChemRisk, 9999 Carver Road, Suite 125, Blue Ash, OH 45242 USA; 3 Division of Developmental Biology, Cincinnati Children’s Hospital Medical Center, 3333 Burnet Ave., Cincinnati, OH 45229 USA; 4 Department of Pediatrics, University of Cincinnati College of Medicine, Cincinnati, OH 45267 USA

**Keywords:** PFOA, Zebrafish, In vivo, Neutrophil, Chemotaxis, Lethal concentration in 50% of embryos (LC_50_), Immunotoxicity, In situ hybridization, Wounding, *Myeloperoxidase*

## Abstract

**Objective:**

Perfluorooctanoic acid (PFOA) is a ubiquitous environmental contaminant and a known immune suppressant in humans and experimental animal models. Studies on PFOA have focused on suppression of the adaptive immune response; however, little is known of the impact on innate immunity, especially during embryogenesis. Therefore, we utilized the zebrafish chemotaxis assay coupled with in situ hybridization for *myeloperoxidase* expression to determine the effects of PFOA exposure on neutrophil migration in the developing zebrafish embryo. Zebrafish embryos are a well-established in vivo model that exhibit high homology with the development of human innate immunity.

**Results:**

Treatment of zebrafish with increasing concentrations of PFOA identified the lethal concentration in 50% of the embryos (LC_50_) to be 300 mg/L. Utilizing the zebrafish chemotaxis assay, this study showed that wounding induced significant neutrophil migration to the site of injury, and that neutrophil number in the wound region was significantly reduced in response to 48-h PFOA exposure (well below doses causing acute mortality). This study demonstrates that the developing embryo is sensitive to PFOA exposure and that PFOA can modify the innate immune system during embryonic development. These results lay the groundwork for future investigation on the mechanisms underlying PFOA-induced developmental immunotoxicity.

## Introduction

Immunotoxicity from exposure to environmental chemicals is an emerging concern, as the prevalence of human disorders with mechanistic roots in immune function continues to increase [1]. Many xenobiotics appear to modulate the immune system at concentrations well below those tested in traditional toxicology studies, possibly contributing to immune-related diseases [1, 2]. Particularly relevant to disease susceptibility is disruption of innate immunity signaling pathways and inflammatory responses, yet the effects of environmental toxicants on innate immunity parameters, especially during early life stages, is not well studied [1].

Perfluorooctanoic acid (PFOA) is ubiquitous in the environment and is detectable and persistent in human serum [3–5]. PFOA was recently “*presumed to be an immune hazard to humans*” by the National Toxicology Program [3] based on data in experimental animals [6–10], and humans [11, 12]. Multiple human studies reported an association of maternal PFOA serum- or plasma-concentrations with decreased antibody response to immunizations in children [11, 12]. These studies suggest that PFOA causes suppression of the adaptive immune system following early life exposures. In contrast, few studies have investigated the effects of PFOA exposure on innate immunity in vivo or during embryonic development. To our knowledge, the developmental immunotoxicity of PFOA has not been well studied, even though developmental processes are sensitive to PFOA [5]. Therefore, the overall goal of this project was to investigate the effects of 48-h PFOA exposure on innate immunity during zebrafish embryogenesis.

While it is difficult to analyze toxicity during early development of mammalian embryos, zebrafish have emerged as an advantageous model system for developmental toxicology. Zebrafish embryos develop externally, and chemical compounds can easily be added to water at defined embryonic stages. Additionally, the cell types and molecular mechanisms in the establishment of innate immunity are well conserved between zebrafish and mammalian embryos [13], and the earliest cells of the immune system to develop include neutrophils. In zebrafish, neutrophils can be specifically identified by the expression of the enzyme myeloid-specific peroxidase (*mpx/mpo*) [14, 15], and the zebrafish chemotaxis assay has been utilized as a model of acute inflammation and hematopoietic cell recruitment in response to tail wounding [16, 17]. Therefore, we developed a wounding assay coupled with in situ hybridization (ISH) targeting *mpx* to identify adverse effects on migrating neutrophils in the developing zebrafish embryo following exposure to PFOA.

## Main text

See Additional file 1: File S1 for detailed methodology.

### PFOA 48-h LC_50_ in Zebrafish Embryos

A 48-h lethal concentration in 50% (LC_50_) of zebrafish embryos was derived to identify concentrations of PFOA causing mortality or overt morphological effects. Zebrafish at 1-h post-fertilization (hpf) were exposed to increasing concentrations of PFOA statically for 48 h, and mortality was analyzed to derive an LC_50_ from concentration–response data.

**Fig. 1:**
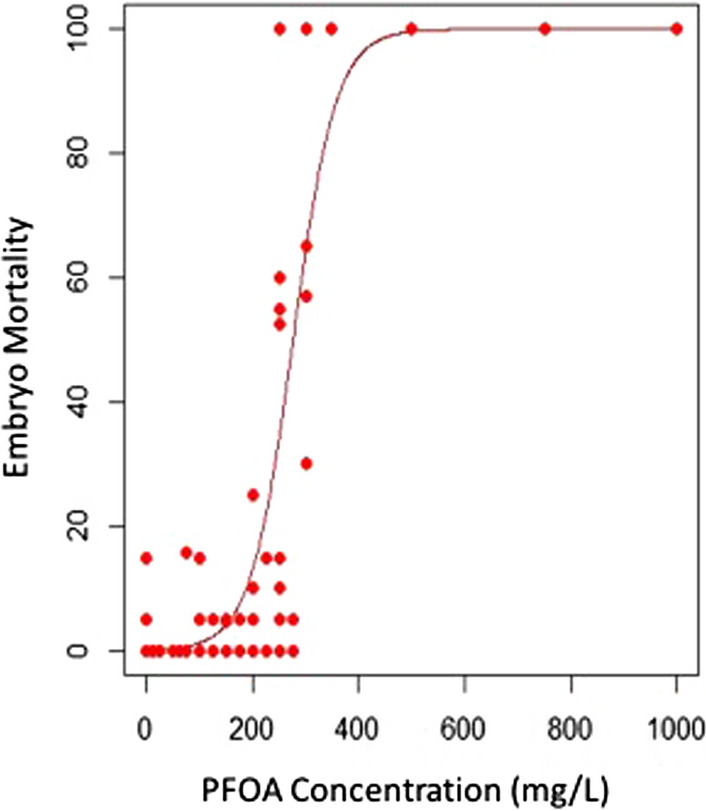
Concentration–response graph showing acute toxicity and the 48-h LC_50_ of PFOA in zebrafish embryos. Concentration of PFOA (X-axis) plotted by the probability of mortality (Y-axis) in zebrafish embryos exposed for 48 h resulted in a derived LC_50_ of 300 mg/L using the ecotoxicity package in R. Each data point represents the average of the replicates from one experiment, and data are presented from five repeated experiments using a range of PFOA concentrations. Each concentration was tested a minimum of three times. Note that the high concentrations all had 100% mortality and those data points overlap

There was little background mortality across five replicate experiments (average 2.6%). The concentration–response for PFOA was steep, with the linear range of the curve (from 20–80% mortality) occurring between 200 and 350 mg/L; the LC_50_ was 300 mg/L (Fig. 1). Malformations were seen in embryos exposed to sublethal concentrations of PFOA (≥ 30 mg/L), including bent tail, yolk sac edema, and cardiac edema. However, the concentration ranges tested were not sufficient for the calculation of effective concentrations (EC_50_) for these sublethal endpoints, due to increasing mortality at higher exposures. The derived LC_50_ was used to identify a testable concentration range for the zebrafish chemotaxis assay that was orders of magnitude below levels causing overt mortality or sublethal morphological effects.

### Analytical chemistry

The PFOA test concentrations in the zebrafish chemotaxis assay (control/DMSO, 0.5 mg/L PFOA, and 5.0 mg/L PFOA) was confirmed using the US EPA Method 537. Six additional perfluoroalkyl substances (PFAS) were included in the analysis as potential co-contaminants (See Additional file 1: Table S1). There were no appreciable concentrations of any of the additional tested PFAS compounds, and the measured PFOA concentrations were roughly at nominal concentrations, with 6.16 mg/L for the high concentration (5.0 mg/L) and 0.685 mg/L for the low concentration (0.5 mg/L). Interestingly, there was a trace level of PFOA (0.089 mg/L) detected in the DMSO control. As PFOA is a known historical water contaminant in the area [18], it is possible that PFOA may be present in trace levels in the fish water or was a contaminant in the DMSO.

### Zebrafish chemotaxis assay

To assess the immunotoxic effects of PFOA exposure at sublethal concentrations, the zebrafish chemotaxis assay was utilized (see Additional file 1: File S1 for detailed methods). Briefly, after 48 h exposure to PFOA, embryos were anesthetized and a small transection at the tip of the tail posterior to the caudal vein was made, as described by Elks et al. [17]. Post-wounding, embryos recovered for three hours to facilitate neutrophil recruitment to the wound site, followed by euthanization and preservation. ISH targeting myeloid-specific peroxidase (*mpx*) mRNA was used to stain and image neutrophils, and total neutrophil number was counted and compared across treatments. Only neutrophils that had migrated to the tip of the tail near the wound region were counted. For more detailed methodology, see Additional file 1: File S1.

It was first demonstrated that tail wounding triggered neutrophil recruitment. Neutrophil migration in the wounded/vehicle control group significantly increased (p < 0.001) compared to unwounded/vehicle control, and wounding in the PFOA treatment (0.5 mg/L) significantly induced neutrophil migration (p = 0.014) compared to unwounded/PFOA control (Additional file 1: Figure S1). This control study confirmed the zebrafish chemotaxis assay as a functional assay leading to neutrophil recruitment to the wound site. In this control experiment, a secondary count of all neutrophils was conducted in the trunk and tail region. These data suggest that there was no systemic difference in neutrophil number across treatments (with and without wounding), although a low number of embryos was included in this analysis (Additional file 1: Figure S1).

**Fig. 2:**
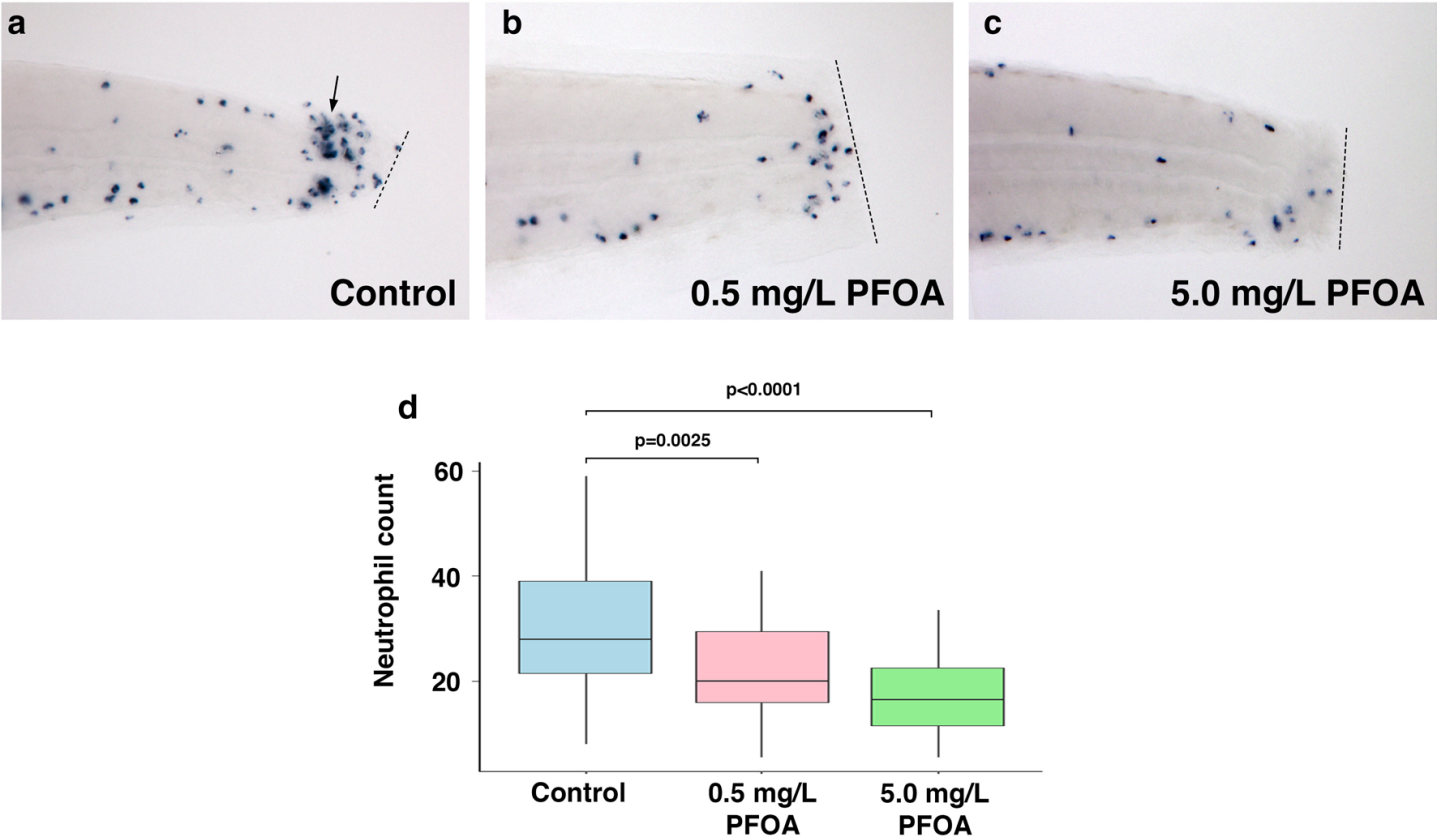
Zebrafish embryos treated with PFOA showed a significant decrease in neutrophil accumulation following wounding. The zebrafish chemotaxis assay showed that PFOA treatment reduced the neutrophil number migrating to the wound site in 48-hpf zebrafish embryos. **a–c**, Representative images of ISH-tagged neutrophils migrating to the wound region (dotted line), where **a** wounded embryos treated with vehicle control (DMSO) (n = 36); **b** wounded embryos treated with PFOA concentration at 0.5 mg/L (n = 35); and **c** wounded embryos treated with PFOA concentration at 5.0 mg/L (n = 31). **d)** The averaged quantification of neutrophil migration in each treatment (error bars represent standard deviation across three replicate experiments). Neutrophil migration in both 0.5 and 5.0 mg/L PFOA was significantly decreased when compared to control using ANOVA, with p-values of 0.0025 and < 0.0001, respectively. The DMSO control never exceeded 0.5% v/v and was constant across all treatments. See Additional file 1: File S1 for detailed methodology

Further zebrafish chemotaxis experiments utilizing 15–20 embryos per treatment and a high (5.0 mg/L) and low (0.5 mg/L) concentration of PFOA were conducted (with a total of three replicate experiments). Concentrations of PFOA (0.5 and 5.0 mg/L) were 60- and 6-fold below the lowest experimental concentration causing sublethal effects, and 400- and 40-fold below the LC_50_, respectively. Results showed a concentration-responsive trend of decreasing neutrophil recruitment with increasing PFOA concentration (Fig. 2). The number of neutrophils at the wound site was reduced 1.4-fold in the 0.5 mg/L PFOA treated embryos and 1.8-fold in the 5.0 mg/L PFOA treated embryos. The effect of PFOA on neutrophils was statistically significant (one-way ANOVA, p = 3.96e-06). The Tukey (multiple comparison) post-hoc analysis showed that both PFOA treatments were different from control, with p = 0.0025 for 0.5 mg/L and p < 0.0001 for 5.0 mg/L. PFOA treatments were not significantly different from each other, although the increase in PFOA concentration from 0.5 to 5.0 mg/L reduced neutrophil accumulation at the wound site by 1.3-fold.

## Discussion

Utilizing the zebrafish chemotaxis assay coupled with in situ hybridization, this study identified an adverse phenotype of reduced neutrophil clustering at the wound site from 48 h exposure to both 0.5 and 5 mg/L PFOA. This demonstrates that the developing embryo is sensitive to PFOA exposure and that PFOA can modify the innate immune system in vivo during embryonic development.

The concentrations of PFOA that resulted in reduced neutrophil accumulation at the wound site in zebrafish were well below those causing lethal effects (48-h LC_50_ = 300 mg/L) and sublethal effects (cardiac edema, yolk sac edema, and bent tail occurring at doses of ≥ 30 mg/L). The LC_50_ is within the range of most values published in the literature (see Additional file 1: Table S2). Differences in LC_50_s derived by the various author groups could be attributed to experimental design including differences in water temperature, duration of experiment, or embryo life stage at the start of the experiment. Additionally, while levels of PFOA found in the sera of the general public are typically below the concentrations tested in this study, occupational exposures can result in serum levels that exceed these concentrations, and little is known about the contribution of other ubiquitous perfluoroalkyl substances to this adverse outcome [5].The suppression of innate immunity identified in our study aligns with effects in the literature showing immunosuppressive effects of PFOA, however most studies have focused on the adaptive immune system and little data investigated innate immunity (for more information on the immunosuppressive effects of PFOA, see reviews by: [3–5, 9]. An in vitro study found that 24-h PFOA exposure significantly reduced placental trophoblast migration at 100 mg/mL [19], supporting a mechanism of altered cellular chemotaxis as opposed to systemic neutropenia. Studies of PFOA on neutrophil numbers in vivo reported an increase in neutrophil number in mice exposed to ≥ 10 mg/kg PFOA for 29 days [10], while another found decreased neutrophils in mice exposed to 40 mg/kg PFOA for 10 days [20]. Neither of these studies covered sensitive developmental life stages, and no published studies were identified for developmental immunotoxicity using the zebrafish model. Therefore, to our knowledge the current study is the first to show inhibition of the innate immune system during sensitive developmental life stages in an in vivo test system.

Results of these studies require further investigation at the molecular and genetic level. Particularly, it is not yet clear if the zebrafish embryos are experiencing neutropenia from PFOA exposure resulting in a reduction in the number of circulating neutrophils (consistent with the systemic leukocytopenia reported in an earlier mouse study [20]), or a specific reduction in neutrophil chemotaxis at the wound site (supported by in vitro data [19] but not tested in vivo). Effects to neutrophil chemotaxis are supported by alteration of inflammatory cytokines and TLR-2 pathways (including MyD88 and NF-kB expression) seen in adult zebrafish exposed to 0.05, 0.1, 0.5, or 1 mg/L PFOA for 21 days [21]. In the current study, the overall neutrophil pool (as evidenced by average neutrophil numbers in the entire trunk and tail region) was similar across treatments which suggest that there is no reduction in overall neutrophil count between PFOA treatments. This adds support to the alternative mechanism of altered neutrophil chemotaxis. However, only a limited number of embryos was analyzed for this endpoint, which may not have been sufficient to observe small changes in the total neutrophil number (Additional file 1: Figure S1). In conclusion, the current study suggests a window of susceptibility for the developing innate immune system from PFOA exposure. These results lay the groundwork for future investigation on the mechanisms underlying PFOA-induced developmental immunotoxicity.

## Limitations

There are a few limitations associated with this study, specifically in relation to the neutrophil chemotaxis assay. These include: (1) the system for counting neutrophils is subjective. However, all images were counted by a semi-blinded researcher (who conducted the experiments) and secondarily by a fully blinded independent researcher, who had no prior knowledge of specific embryo treatments. While the neutrophil counts between the two researchers were different, the statistical significance of the effect was of similar magnitude. (2) Difficulties in producing identical wounds across all embryos could be contributing to high variability in neutrophil number at the wound site. However, while the wounds were not identical, the cut was made similarly for each embryo. The repeated ability to detect a significant effect across experiments and to find statistical significance even with this high variability suggests the assay is sensitive. (3) Finally, the analysis of the total number of neutrophils was performed in a relatively small number of embryos. It would be beneficial to analyze larger number of embryos in the future to exclude a possibility of a minor reduction, if any, in the overall neutrophil number due to PFOA exposure.

## Supplementary information

**Additional file 1.** Supplementary Data File contains detailed methodology information for studies conducted, and contains Supplemental Tables S1–S2 and Supplemental Figure S1.

## Data Availability

The datasets used and analyzed during the current study are available from the corresponding author(s) on reasonable request.

## Abbreviations

ATSDR: Agency for Toxic Substances and Disease Registry
ANOVA: Analysis of variance
CCHMC: Cincinnati Children’s Hospital Medical Center
DMSO: Dimethyl sulfoxide
EC_50_: Effective concentrations
Hpf: Hours post fertilization
ISH: In situ hybridization
LC_50_: Lethal concentration in 50%
LPS: Lipopolysaccharide
mg/L: Milligrams per liter
MRL: Minimal Risk Level
*mpx/mpo*: Myeloid-specific peroxidase/myeloperoxidase
NTP: National Toxicology Program
OECD: Organization for Economic Cooperation and Development
PFAS: Perfluoroalkyl substances
PFBSA: Perfluorobutanesulfonic acid
PFHA: Perfluoroheptanoic acid
PFHSA: Perfluorohexanesulfonic acid
PFOOA: Perfluorononanoic acid
PFOSA: Perfluorooctanesulfonic acid
PFOA: Perfluorooctanoic acid
PTU: 1-Phenyl-2-thiourea
US EPA: United States Environmental Protection Agency

## References

[1] Hartung T, Corsini E (2013). Immunotoxicology: challenges in the 21st century and in vitro opportunities. Altex..

[2] Dietert RR, Luebke RW. The Environment-immune route to chronic disease. In: Dietert RR, Luebke RW, editors. Immunotoxicity, Immune Dysfunction, and Chronic Disease, Molecular and Integrative Toxicology. Humana Press; 2015. p. 31-47.

[3] National Toxicology Program (NTP). NTP Monograph. Immunotoxicity Associated with Exposure to Perfluorooctanoic Acid or Perfluorooctane Sulfonate. National Institute of Environmental Health Sciences, National Institutes of Health. U.S. Department of Health and Human Services; 2016.

[4] DeWitt JC, Blossom SJ, Schaider LA (2019). Exposure to per-fluoroalkyl and polyfluoroalkyl substances leads to immunotoxicity: epidemiological and toxicological evidence. J Expo Sci Environ Epidemiol.

[5] Agency for Toxic Substances and Disease Registry (ATSDR). Toxicological Profile for Perfluoroalkyls. Draft for Public Comment. Division of Toxicology and Human Health Sciences; 2018.

[6] Yang Q, Abedi-Valugerdi M, Xie Y, Zhao XY, Moller G, Nelson BD, DePierre JW (2002). Potent suppression of the adaptive immune response in mice upon dietary exposure to the potent peroxisome proliferator, perfluorooctanoic acid. Int Immunopharmacol.

[7] Dewitt JC, Copeland CB, Strynar MJ, Luebke RW (2008). Perfluorooctanoic acid-induced immunomodulation in adult C57BL/6J or C57BL/6N female mice. Environ Health Perspect.

[8] DeWitt JC, Copeland CB, Luebke RW (2009). Suppression of humoral immunity by perfluorooctanoic acid is independent of elevated serum corticosterone concentration in mice. Toxicol Sci.

[9] DeWitt JC, Williams WC, Creech NJ, Luebke RW (2016). Suppression of antigen-specific antibody responses in mice exposed to perfluorooctanoic acid: role of PPARα and T- and B-cell targeting. J Immunotoxicol.

[10] Loveless SE, Hoban D, Sykes G, Frame SR, Everds NE (2008). Evaluation of the immune system in rats and mice administered linear ammonium perfluorooctanoate. Toxicol Sci.

[11] Grandjean P, Andersen EW, Budtz-Jorgensen E, Nielsen F, Molbak K, Weihe P, Heilmann C (2012). Serum vaccine antibody concentrations in children exposed to perfluorinated compounds. J Am Med Assoc.

[12] Granum B, Haug LS, Namork E, Stolevik SB, Thomsen C, Aaberge IS, van Loveren H, Lovik M, Nygaard UC (2013). Pre-natal exposure to perfluoroalkyl substances may be associated with altered vaccine antibody levels and immune-related health outcomes in early childhood. J Immunotox.

[13] Forn-Cuni G, Varela M, Pereiro P, Novoa B, Figueras A (2017). Conserved gene regulation during acute inflammation between zebrafish and mammals. Nat Sci Reports.

[14] Lieschke GJ, Oates AC, Crowhurst MO, Ward AC, Layton JE (2001). Morphologic and functional characterization of granulocytes and macrophages in embryonic and adult zebrafish. Blood.

[15] Mathias JR, Perrin BJ, Liu TX, Kanki J, Look AT, Huttenlocher A (2006). Resolution of inflammation by retrograde chemotaxis of neutrophils in transgenic zebrafish. J Leukoc Bio.

[16] Falenta K, Rodaway A, Jones GE, Wells CM (2013). Imaging haematopoietic cells recruitment to an acute wound in vivo identifies a role for c-Met signalling. J Microscopy.

[17] Elks PM, Loynes CA, Renshaw SA (2011). Measuring inflammatory cell migration in the zebrafish. Methods Mol Biol.

[18] Herrick RL, Buckholz J, Biro FM, Calafat AM, Ye X, Xie C, Pinney SM (2017). Polyfluoroalkyl substance exposure in the Mid-Ohio River Valley, 1991–2012. Environ Pollut.

[19] Szilagyi JT, Freedman AN, Kepper SL, Keshava AM, Bangma JT, Fry RC (2020). Per- and polyfluoroalkyl substances differentially inhibit placental trophoblast migration and invasion in vitro. Toxicol Sci.

[20] Qazi MR, Bogdanska J, Butenhoff JL, Nelson BD, DePierre JW, Abedi-Valugerdi M (2009). High-dose, short-term exposure of mice to perfluorooctanesulfonate (PFOS) or perfluorooctanoate (PFOA) affects the number of circulating neutrophils differently, but enhances the inflammatory responses of macrophages to lipopolysaccharide (LPS) in a similar fashion. Toxicol.

[21] Zhang H, Fang W, Wang D, Gao N, Ding Y, Chen C (2014). The role of interleukin family in perfluorooctanoic acid (PFOA)-induced immunotoxicity. J Hazard Mater.

[22] United States Environmental Protection Agency (US EPA). OPPTS 850.1400 Fish Early-Life Stage Toxicity Test. Ecological Effects Test Guidelines Prevention, Pesticides and Toxic Substances. 1996; EPA 712-C-96-121.

[23] Jowett T (1999). Analysis of protein and gene expression. Methods Cell Biol.

[24] Bennett CM, Kanki JP, Rhodes J, Liu TX, Paw BH, Kieran MW, Langenau DM, Delahaye-Brown A, Zon LI, Fleming MD, Look AT (2001). Myelopoiesis in the zebrafish, Danio rerio. Blood.

[25] Rainieri S, Conlledo N, Langerholc T, Madorran E, Sala M, Barranco A (2017). Toxic effects of perfluorinated compounds at human cellular level and on a model vertebrate. Food Chem Toxicol.

[26] Weiss-Errico MJ, Berry JP, O'Shea KE (2017). Beta-cyclodextrin attenuates perfluorooctanoic acid toxicity in the zebrafish embryo model. Toxics..

[27] Zheng XM, Liu HL, Shi W, Wei S, Giesy JP, Yu HX (2011). Effects of perfluorinated compounds on development of zebrafish embryos. Environ Sci Pollut Res Int.

[28] Hagenaars A, Vergauwen L, De Coen W, Knapen D (2011). Structure-activity relationship assessment of four perfluorinated chemicals using a prolonged zebrafish early life stage test. Chemosphere.

[29] Godfrey A, Abdel-Moneim A, Sepulveda MS (2017). Acute mixture toxicity of halogenated chemicals and their next generation counterparts on zebrafish embryos. Chemosphere.

[30] Stengel D, Wahby S, Braunbeck T (2018). In search of a comprehensible set of endpoints for the routine monitoring of neurotoxicity in vertebrates: sensory perception and nerve transmission in zebrafish (*Danio rerio*) embryos. Environ Sci Pollut Res Int.

[31] Corrales J, Kristofco LA, Steele WB, Saari GN, Kostal J, Williams ES, Mills M, Gallagher EP, Kavanagh TJ, Simcox N, Shen LQ, Melnikov F, Zimmerman JB, Voutchkova-Kostal AM, Anastas PT, Brooks BW (2017). Toward the design of less hazardous chemicals: exploring comparative oxidative stress in two common animal models. Chem Res Toxicol.

[32] Ulhaq M, Carlsson G, Orn S, Norrgren L (2013). Comparison of developmental toxicity of seven perfluoroalkyl acids to zebrafish embryos. Environ Toxicol Pharmacol.

[33] Organization for Economic Cooperation and Development (OECD). Test No. 236: Fish Embryo Acute Toxicity (FET) Test. OECD Guidelines for the Testing of Chemicals 2013, Section 2. Paris: OECD Publishing. https://doi.org/10.1787/9789264203709-en.

