# Supplementary material for: Exposure to perfluorooctanoic acid (PFOA) decreases neutrophil migration response to injury in zebrafish embryos
Source: BMC Res Notes. 2020 Aug 31;13:408. doi: 10.1186/s13104-020-05255-3 (PMC7460781; doi:10.1186/s13104-020-05255-3)
Supplement: Supplementary file 1 — Additional file 1. Supplementary Data File contains detailed methodology information for studies conducted, and contains Supplemental Tables S1–S2 and Supplemental Figure S1. [file 13104_2020_5255_MOESM1_ESM.docx]

**Supplemental Files (S1)**

**Title:** Exposure to Perfluorooctanoic Acid (PFOA) Decreases Neutrophil Migration Response to Injury in Zebrafish Embryos

**Authors:** Pecquet, A.M., Maier, M.A., Kasper, S., Sumanas, S., Yadav, J.

**Detailed Methods**

*Zebrafish Husbandry*

The transgenic zebrafish line *Tg(mpx:GFP)^uwm1^* (Mathias et al., 2006) used in this study was maintained at the Cincinnati Children’s Hospital Medical Center (CCHMC) aquatics facility. In this model, neutrophils express green fluorescent protein under the control of the *myeloperoxidase* promoter (*mpo/mpx*), which allowed the *in vivo* tracking of neutrophil migration during the acute inflammatory response. Zebrafish were kept at 28^◦^C with a 14:10-hour light:dark cycle in a recirculating tank system (Aquatic Habitats, water pH 7.2–7.6, salinity 0.03%–0.04%). Breeding was conducted in small breeding tanks with a 2:1 male:female ratio. Tank dividers were pulled each morning after facility lights came on. Zebrafish were allowed to spawn for one hour, at which time the eggs were collected. Fertilized embryos were collected in clean Petri dishes and maintained in fish water containing 60 mg/L “Instant Ocean” salt and 2 mg/L methylene blue.

*Chemicals*

Perfluorooctanoic acid (PFOA) was obtained as a generous gift from Dr. Shouxiong Huang, Department of Environmental and Public Health Sciences, University of Cincinnati, Cincinnati OH. A stock solution of 1 mg/mL PFOA was prepared in dimethyl sulfoxide (DMSO) and diluted further into final concentrations of 0.5 mg/L and 5.0 mg/L PFOA in fish water (as described above but without the addition of methylene blue) for the zebrafish chemotaxis assay. An equal volume of DMSO (5 μL/ml) served as the vehicle control for all experiments and the final concentration of DMSO never exceeded 0.5% v/v. The LC_50_ experiments used PFOA directly dissolved into fish water without DMSO, following heating on a stir plate at a low heat for 15 minutes. To inhibit melanin formation (pigmentation), 0.003% 1-phenyl-2-thiourea (PTU) (Sigma-Aldrich) was added to the fish treatment water after 24 hours. 1.6 mg/L Tricaine (Sigma-Aldrich) was utilized to anesthetize embryos.

*Zebrafish LC_50_ determination*

Lethal concentration in 50% of embryos (LC_50_) experiments (48-hour) were conducted to assess lethality of PFOA to zebrafish embryos and identify concentrations causing overt sublethal malformations. Briefly, embryos were selected at the 1-hour post fertilization (hpf) developmental stage. Two guidance documents, the OECD Fish Embryo Toxicity Test Guideline 236 (OECD, 2013) and the US EPA Fish Early-life Stage Toxicity Test Guideline 210 (US EPA, 1996) were utilized for development of the protocol in relation to PFOA testing concentrations, number of embryos used per treatment, developmental stage of embryos, and duration of exposure. Embryos were exposed to varying concentrations of PFOA in five replicate experiments, ranging from 0 – 1000 mg/L PFOA. The exposures were static (non-renewal) for 48-hours at 28°C. Embryos were assessed at 3, 5, 24, and 48 hours for mortality, while sublethal effects (including skeletal malformations, cardiac edema, yolk sac edema) and mortality were assessed at the end of the 48-hour exposure period. Each experiment was conducted with seven PFOA concentrations, three replicates per concentration, and 20 embryos per replicate.

*Analytical chemistry*

Analytical chemistry of the test samples was provided by Pace Analytical Laboratories (Jacksonville, FL) using the US EPA Test Method 537. Samples taken from the zebrafish chemotaxis assay experiment included: fish water (DMSO control), low concentration (0.5 mg/L PFOA), and high concentration (5.0 mg/L PFOA). The laboratory provided chain of custody-labeled collection vials of 500 mL polypropylene containers with polypropylene screw caps containing 5.0 g/L Trizma (as a buffer) in a refrigerated cooler and were shipped overnight to the laboratory.

*Zebrafish chemotaxis assay*

The zebrafish chemotaxis assay was utilized to assess the effects of PFOA exposure at sublethal concentrations on neutrophil migration in response to wounding using a tail transection protocol.

Following breeding for one hour (as described above), embryos were scanned using a dissecting microscope to identify those at the 4-cell developmental stage (1-hpf). Zebrafish at 1-hpf were exposed for 24 hours to vehicle control (DMSO at 5 µL/mL), 0.5 mg/L, or 5.0 mg/L PFOA in fish water. Embryos (5-20, as indicated in the Results Section) were exposed per treatment. Exposures were conducted in a covered 24-well plate in a rotisserie incubator. The rotisserie arm was set to gently oscillate the water and the incubation temperature was maintained at 28^◦^C.

Following 24 hours exposure, embryos were checked for mortality and deformities. Dead embryos (if present) were removed and counted. PTU (0.003%) was added to the remaining viable embryos to inhibit melanin formation (pigmentation), and the embryos were incubated for an additional 24 hours in their respective PFOA or control treatments.

At the end of the 48-hour period, embryos were manually dechorionated using sharpened tweezers and transferred to clean fish water. Embryos were anesthetized using 1.6 mg/L Tricaine and the tip of the tail was wounded using a modified tail transfection protocol from Elks et al. (2011). Briefly, dechorionated embryos were placed onto a piece of tape on a petri dish and excess water was removed with a pipette. Embryos were oriented with the tails facing the same direction, and the petri dish was placed under a dissection microscope for tail transection. Wounding was performed using a microsurgical knife to make a small transection at the tip of the tail, posterior to the caudal vein without clipping the vein. Post-wounding, embryos were transferred to clean fish water to recover and allow neutrophil response to the wound for three hours, euthanized using Tricaine, and immediately preserved in BT-fix overnight at 4^◦^C on a nutator. After 24 hours, embryos were dehydrated in ethanol using serial dilutions (30, 50, 70, 100% dilutions) and stored at -20^◦^C.

*In Situ Hybridization*

Embryos were rehydrated using serial dilutions and *in situ* hybridization (ISH) was performed as previously described (Jowett, 1999). Briefly, embryos were washed in PBT and briefly incubated with proteinase K. Next, pre-hybridization wash was followed by overnight (18 hours) hybridization with the *mpx* (neutrophil specific) riboprobe (Bennett et al., 2001) labeled with digoxigenin-UTP (Roche). On day 2, embryos were washed in pre-hybridization buffer and incubated overnight with pre-absorbed digoxigenin antibody. On day 3, embryos were washed in PBT, transferred to developing solution, and allowed to develop for 3 hours. Following developing, embryos were dehydrated back into 100% ethanol using serial dilutions and stored at 4^◦^C until imaging.

For imaging, embryos were rehydrated and mounted laterally on slides using 0.6% low melting point agarose (Sigma-Aldrich). Images were captured using a 10× objective on an AxioImager Z1 (Zeiss) compound microscope with an Axiocam ICC3 color camera (Zeiss). Tail portion (from yolk sac to tip of tail) images of the stained neutrophils in multiple focal plans were captured individually and combined using the Extended Focus (Z-stack) module within Axiovision 4.9 software (Zeiss). For the control experiment (shown in Supplemental Fig. S1), we first counted the neutrophils that were present in the wound region/tip of the tail. In this control experiment (Suppl. Fig. S1f), we additionally counted all neutrophils present in the tail and trunk region from the tip of the tail including the yolk extension to the posterior edge of the yolk. In the experiments shown in Fig. 2, counts were conducted only of neutrophils that had migrated to the tip of the tail in the wound region. Total number of stained neutrophils migrated to the tail wound site were determined by a semi-blind count (AMP) and confirmed by a fully blind count (EF). In the semi-blind count, the images were assigned random numbers, resorted, and counted (AMP). In the fully blind count, the researcher (EF) had no previous knowledge of treatments and counted images assigned random numbers and sorted, as to reduce bias in the counting procedure. Counts from the two researchers were averaged. One-way ANOVA was performed to identify treatment differences and Tukey test was used to assess statistical significance between experimental groups individually.

*Statistical analysis*

Statistical differences between treatments was analyzed using ANOVA in R package with Tukey (multiple comparison) post-hoc analysis. R ecotoxicity package was used to generate LC_50_ data by plotting concentration by mortality incidence.

**Supplemental Tables**

**Table S1. Analytical chemistry for PFOA to assess concentration and purity using US EPA Test Method 537.**

|  | **Analyte (mg/L)** | | | | | |
| --- | --- | --- | --- | --- | --- | --- |
| **Sample** | **PFOA** | PFBSA | PFHA | PFHSA | PFOOA | PFOSA |
| **DMSO Control** | **0.089** | ND | ND | ND | ND | ND |
| **PFOA high concentration (5.0 mg/L)** | **6.16** | ND | 0.00097 | ND | 0.00054 | ND |
| **PFOA low concentration (0.5 mg/L)** | **0.685** | ND | 0.000065 | ND | ND | ND |

ND = Not detected at the method detection limit, which varies per chemical but ranges from 0.0013 to 0.057 µg/L; Bolded column represents PFOA, the test chemical used in the experiments. PFOA = Perfluorooctanoic acid; PFBSA = Perfluorobutanesulfonic acid; PFHA = Perfluoroheptanoic acid; PFHSA = Perfluorohexanesulfonic acid; PFOOA = Perfluorononanoic acid; PFOSA = Perfluorooctanesulfonic acid

**Table S2. LC_50_s in zebrafish embryos exposed to PFOA as determined in different studies.**

| **Study Citation** | **Study Duration** | **LC_50_** |
| --- | --- | --- |
| Current study | 2 days/48 hours | 300 mg/L |
| Rainieri et al., 2017 | 2 days/48 hours | >500 mg/L |
| Weiss-Errico et al., 2017 | 2 days/48 hours | 50 mg/L |
| Zheng et al., 2012 | 3 days/72 hours | 262 mg/L |
| Hagenaars et al., 2011 | 4 days/96 hours | >500 mg/L |
| Godfrey et al., 2017 | 4 days/96 hours | 473 mg/L |
| Stengel et al., 2018 | 4 days/96 hours | 759 mg/L |
| Corrales et al., 2016 | 4 days/96 hours | 24.6 mg/L |
| Ulhaq et al., 2013 | 6-days/144 hours | 430 mg/L |

**Supplemental Figure**


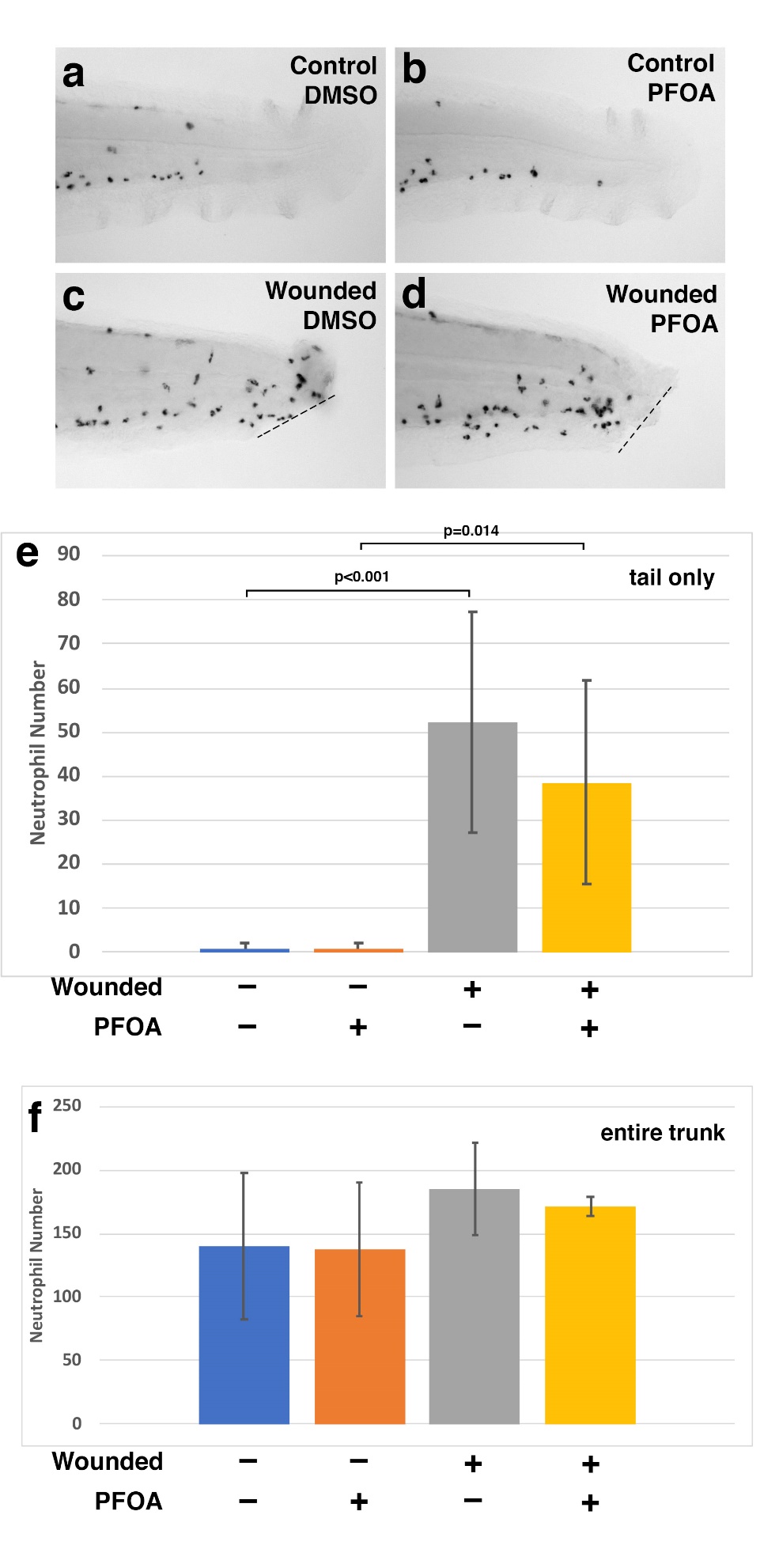


**Figure S1. The zebrafish chemotaxis assay showed that wounding induced neutrophil migration to the tail region.** Proof-of-concept study results showed that wounding increases neutrophil recruitment to the tail region in 48-hpf zebrafish embryos. **a-d,** Representative images of ISH-tagged neutrophils migrating to the wound region (dotted line), where **a)** unwounded embryo treated with vehicle control (DMSO at 0.5% v/v); **b)** unwounded embryo treated with PFOA concentration at 0.5 mg/L; **c)** wounded embryo treated with vehicle control; and **d)** wounded embryo treated with PFOA concentration at 0.5 mg/L. **e)** Quantification of neutrophil number at the tail region in each treatment group. n = 5 embryos/treatment. A significant increase in neutrophils (p <0.001) was observed in the vehicle control treated embryos when wounded as compared to unwounded. The difference in neutrophils in 0.5 mg/L PFOA treated embryos was significant at p = 0.014 when wounded as compared to unwounded. **f)** The total neutrophil number in the trunk and tail region. n = 5 embryos/treatment. Error bars represent standard deviations. There were no significant differences between any of the groups analyzed (p>0.05), suggesting that there were no overall changes in neutrophil number after PFOA treatment.
